# Acute Jejunal Diverticulitis Induced by a Malignant Gastrointestinal Neuroectodermal Tumor: A Case Report

**DOI:** 10.7759/cureus.75127

**Published:** 2024-12-04

**Authors:** Meshael S Albahli, Shoag J Albugami, Nourah I Alabdulaaly, Raghad Tarabishi, Haider A Alshurafa, Khaled Altoukhi

**Affiliations:** 1 General Surgery, Prince Sultan Military Medical City, Riyadh, SAU; 2 Hepatobiliary Surgery, Prince Sultan Military Medical City, Riyadh, SAU; 3 Surgical Oncology, Prince Sultan Military Medical City, Riyadh, SAU

**Keywords:** acute abdomen, acute diverticulitis, gastrointestinal tumor, jejunum, neuroectodermal tumor

## Abstract

Small intestinal diverticula induced by malignant mesenchymal tumors are extremely rare clinical entities. We present the case of a 46-year-old female who reported a one-week history of worsening generalized abdominal pain accompanied by constipation. A computed tomography (CT) scan of her abdomen revealed an ulcerative mass at the proximal/midjejunal junction, suggestive of jejunal diverticulitis. She initially underwent diagnostic laparoscopy, which was converted to an open procedure for resection of the jejunal mass and side-to-side anastomosis. The final histopathological examination revealed features consistent with a gastrointestinal neuroectodermal tumor.

## Introduction

Small intestinal diverticula are rare clinical entities in which the mucosa and submucosa herniate through the muscular layer of the intestinal wall [1]. These intestinal diverticula are classified as pulsion diverticula and result from intestinal dyskinesia [2]. The condition has a 1-5% prevalence in the general population. Furthermore, the prevalence increases with age, especially in males in the sixth to eighth decades of life [1,2]. Small bowel diverticula are rare, and 80% of cases are found in the jejunum, 15% in the ileum, and 5% in both [3,4]. Chronic symptoms associated with jejunal diverticula (JD) can include abdominal discomfort, a sensation of postprandial fullness, abdominal cramping, and anemia secondary to iron or vitamin B12 deficiency resulting from malabsorption; however, most patients with JD are asymptomatic [4]. Bowel perforation, mechanical obstruction, and hemorrhage are major complications that may present initially in cases of JD. Diagnosing complicated jejunal diverticulosis can be challenging and may not always be accurate, even with high-resolution imaging techniques; in some cases, a diagnostic laparotomy may be necessary [3,5]. Acute diverticulitis occurs when these diverticula become inflamed or infected. This condition typically manifests with severe, vague abdominal pain, nausea, fever, and altered bowel habits. The incidence of diverticulitis increases with age. Other risk factors include obesity, smoking, excessive alcohol consumption, a sedentary lifestyle, high-fat, low-fiber diets, and the use of certain medications, such as steroids, opioids, and non-steroidal anti-inflammatory drugs (NSAIDs) [6]. Surgical intervention should be considered before severe complications arise [3]. In this article, we present a case of an adult female patient who was diagnosed with jejunal diverticulitis.

## Case presentation

The patient is a 46-year-old female who presented with a one-week history of generalized abdominal pain, which worsened one day before her visit. The abdominal pain was accompanied by nausea, vomiting, fever, sweating, and constipation. She reported similar symptoms intermittently, starting eight months prior. There was no history of rectal bleeding, hematemesis, or unintentional weight loss. Her family history was notable for her mother, who had been treated for pulmonary tuberculosis. Upon presentation, her vital signs were as follows: blood pressure of 132/80 mmHg; respiratory rate of 20 bpm; oral temperature of 36.9°C; and heart rate between 87 and 98 bpm. Physical examination revealed a soft, nondistended abdomen with mild tenderness throughout, but there were no signs of rebound tenderness or peritoneal irritation. Laboratory tests revealed a normal complete blood count and electrolyte levels.

**Figure 1:**
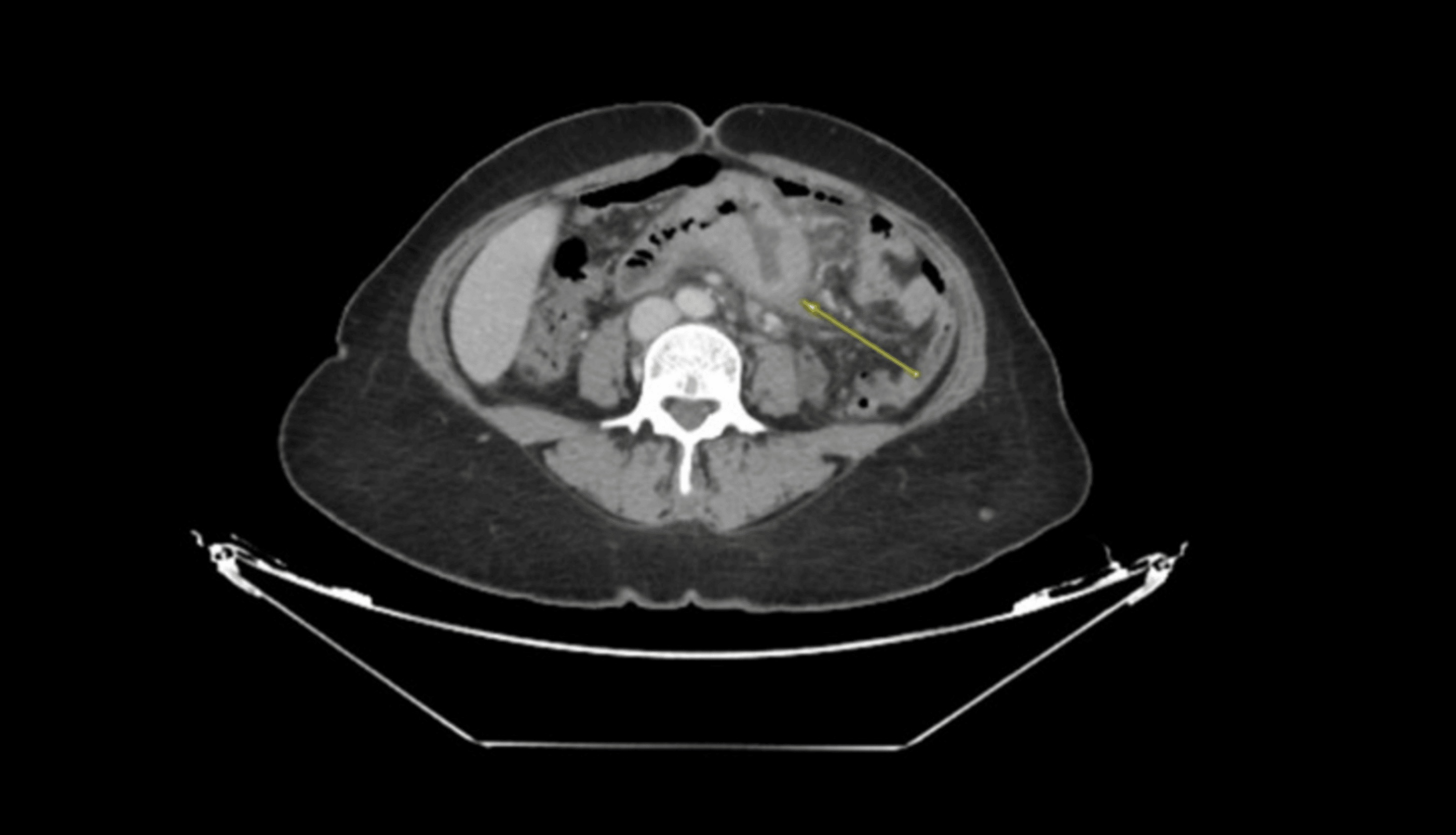
Computed tomography scan showing the thickening and enhancement of the proximal middle jejunum (yellow arrow)

**Figure 2:**
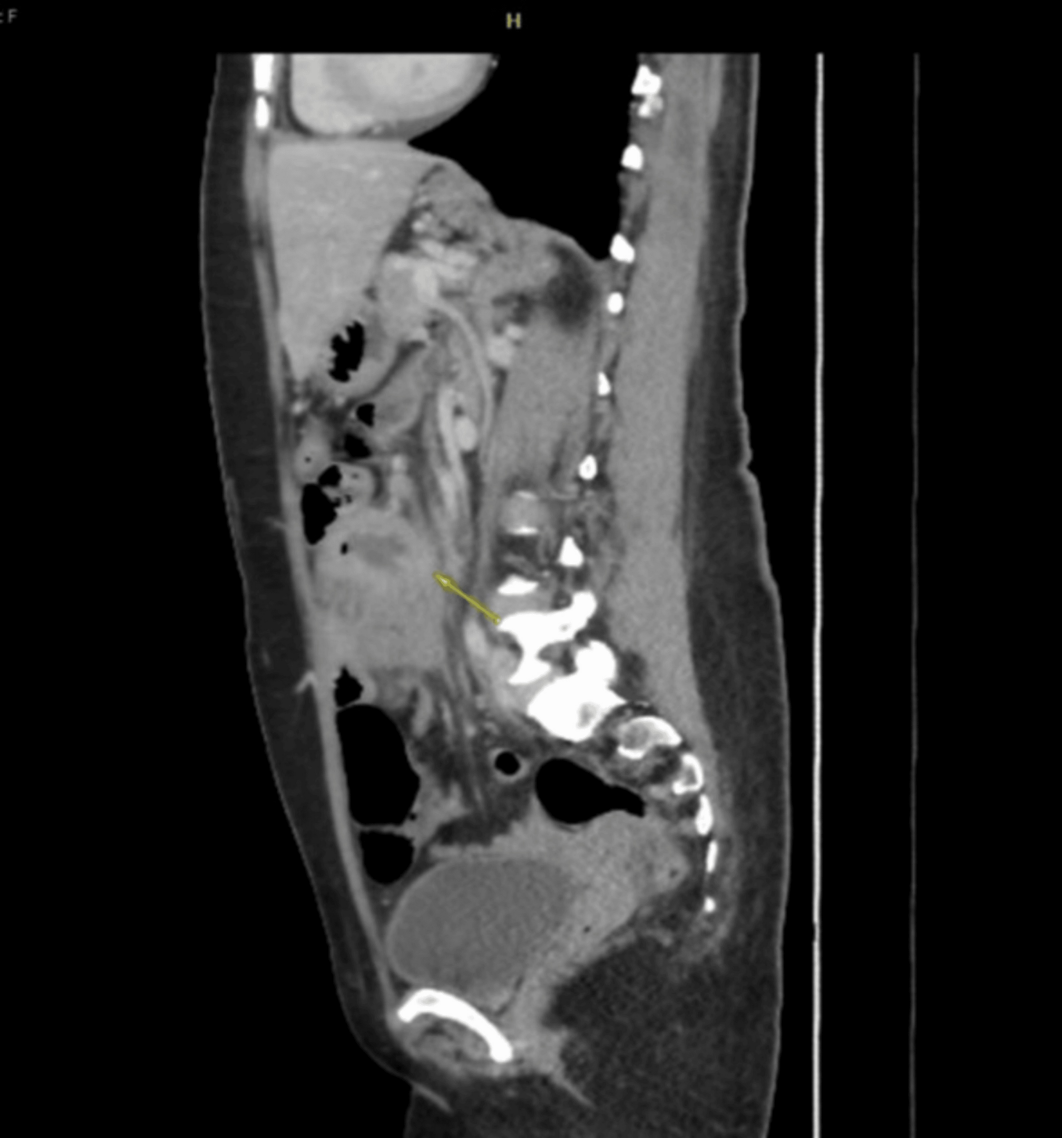
Computed tomography scan showing the sagittal view of thickening and enhancement of the proximal midjejunum (yellow arrow)

A computed tomography (CT) scan of the abdomen revealed a short segment of circumferential thickening and enhancement at the proximal midjejunal junction, measuring approximately 3.2 cm in length and located approximately 15 cm from the duodenojejunal junction. This finding was associated with a focal defect in the bowel wall, resulting in a 3.9 × 3.2 cm distance. The outpouch contained high-density fluid, likely representing acute blood, along with irregularities suggestive of ulceration and impending perforation. Significant fat stranding and free fluid were observed around the outpouch, tethering adjacent small bowel loops, as were multiple enlarged mesenteric lymph nodes, the largest measuring 0.7 cm. Prominent right common iliac lymph nodes measuring 0.6 cm were also noted. There was no pneumoperitoneum, and no contrast extravasation was suggested for active bleeding or perforation. Pneumatosis intestinalis was absent (Figures 1-2).

**Figure 3:**
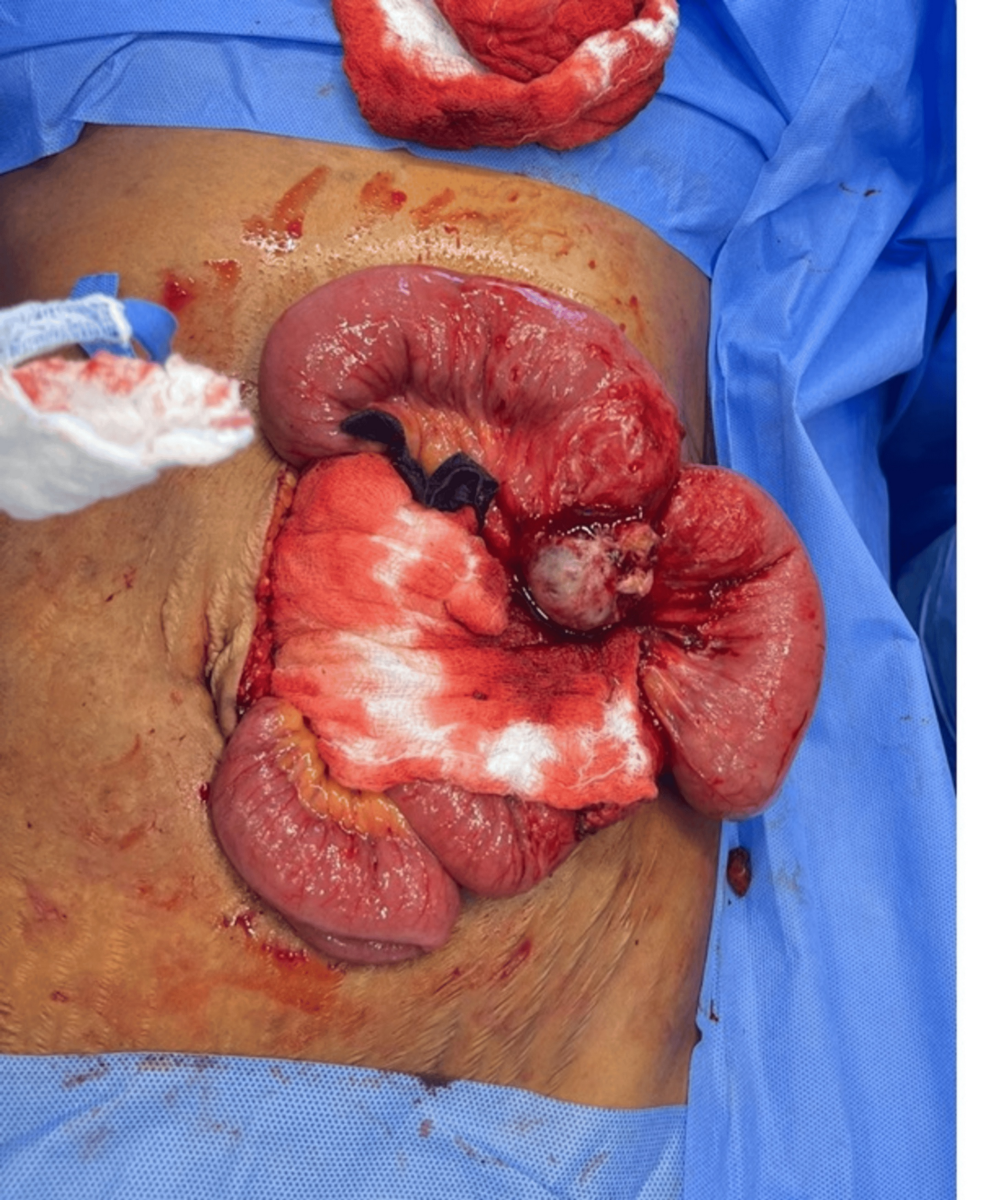
Operative view showing a jejunal mass arising from the root of the mesentery and adhering to the small bowel. The jejunal mass/wall thickening with outpouring was identified without clear perforation or spillage

**Figure 4:**
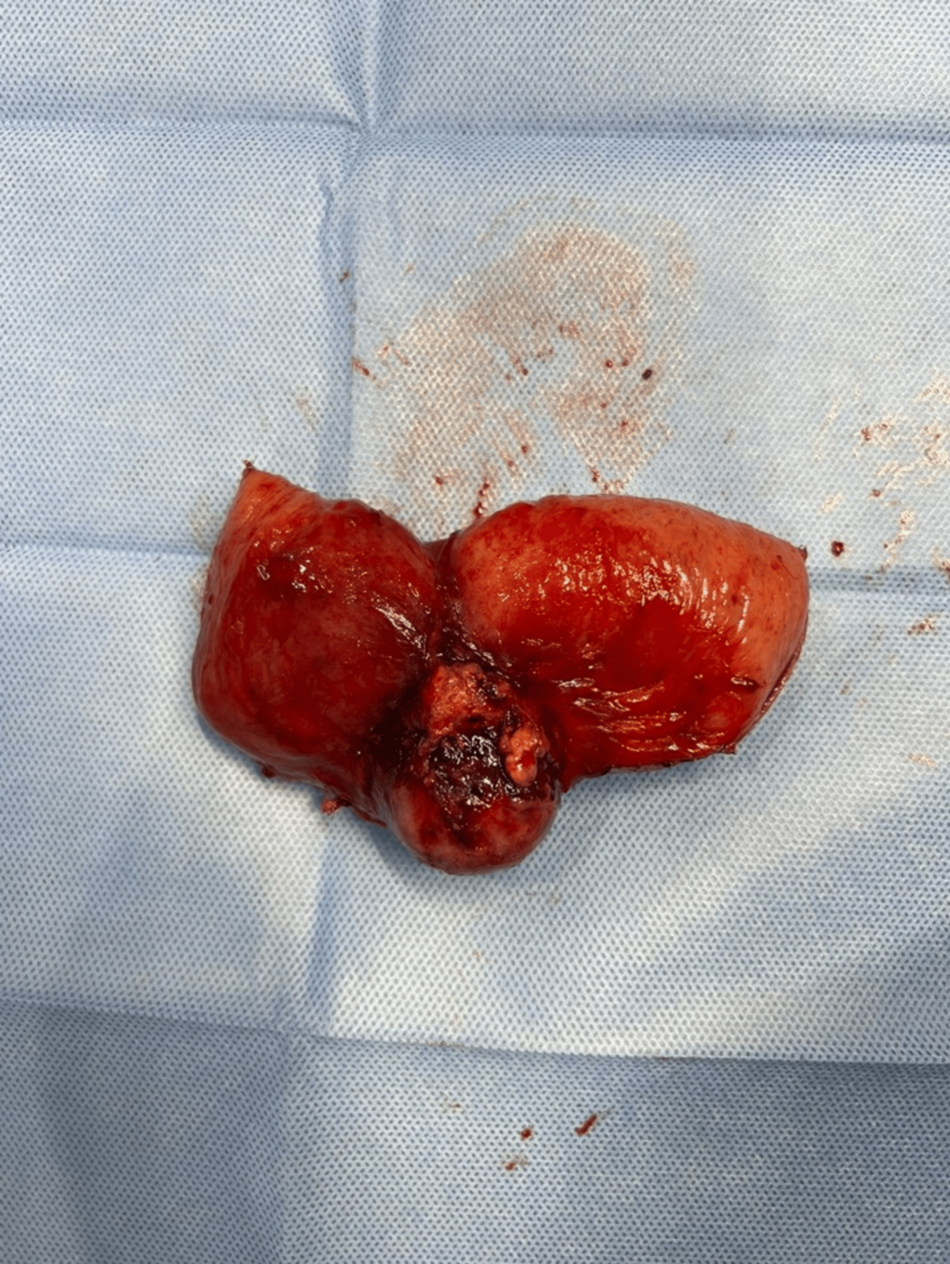
Resected jejunal mass

The differential diagnosis included gastrointestinal stromal tumor (GIST), neuroendocrine tumor, lymphoma, and jejunal diverticulitis. The patient was admitted to the general surgery service, kept nil per os, and started on intravenous fluids. She was administered antibiotics (intravenous piperacillin sodium/tazobactam sodium). Further investigation via positron emission tomography (PET) revealed circumferential thickening of the midjejunal wall with high 18-fluoro-deoxyglucose (FDG-PET) uptake. Multiple enlarged mesenteric and right common iliac lymph nodes exhibited variable FDG-PET uptake, indicating a high suspicion of malignant lesions. Surgical intervention was scheduled, and the patient underwent diagnostic laparoscopy, which was converted to laparotomy. Intraoperative findings revealed a jejunal mass adhering to the small bowel, forming a phlegmon. A jejunal mass with outpouching was identified, but there was no clear perforation or spillage (Figure 3). Segmental resection was performed via a side-to-side anastomosis (Figure 4).

The postoperative course was unremarkable; the patient gradually resumed her diet, had regular bowel movements, and was discharged in good condition on postoperative day 3. Histopathological examination of the small bowel segment revealed a smooth gray-tan serosa with serosal outpouching. The mass measured 3.5 × 3.0 × 2.5 cm and was filled with hemorrhagic autolyzed material. The lesion was located 4.0 cm from the proximal and distal margins abutting the serosa. No gross evidence of perforation, abscess, or other lesions was found. Immunohistochemical analysis of the surgical specimens revealed that the tumor cells were positive for S100 protein and SOX10 but negative for CKAE1/AE3, inhibin, PAX8, calretinin, actin, desmin, myogenin, MyoD1, CD34, CD117, DOG1, MelanA, HMB45, chromogranin, synaptophysin, CD20, CD3, and CD45. Thus, the final histopathological and immunohistochemical findings supported a diagnosis of gastrointestinal neuroectodermal tumors (GNETs). Following discharge, the patient was followed in the clinic and reported no active complaints. A discussion by the tumor board regarding the pathology results was performed, with a final decision for observation and follow-up at six months with a PET-CT scan to rule out recurrence.

## Discussion

Malignant mesenchymal tumors, such as malignant GNETs, are extremely rare primary malignant mesenchymal tumors, and the induction of acute jejunal diverticulitis makes them much rarer, which makes it a complicated disease that necessitates a thorough comprehension of the underlying pathologies [7,8]. Diverticular disease can result from mesenchymal tumors that appear in the small intestine, including GISTs, various carcinomas, and malignant GNETs [7,9]. Despite being uncommon, jejunal diverticulitis can have major complications, such as perforation, obstruction, peritonitis, and bleeding. Acute jejunal diverticulitis can mimic other more prevalent diseases, such as inflammatory bowel disease or colonic diverticulitis, which makes the clinical setting more complicated [10,11]. Magnetic resonance imaging can help with surgical planning and provide details about the extent of the lesion [12]. Surgical intervention is the treatment of choice for complicated acute jejunal diverticulitis, particularly when the condition is worsened by a mesenchymal tumor [3,9]. A multidisciplinary approach is necessary for the surgical care of small intestine mesenchymal tumors, particularly when there are acute complications such as diverticulitis [11].

## Conclusions

The occurrence of acute jejunal diverticulitis due to a malignant mesenchymal tumor, such as a GNET, presents a complex clinical challenge that necessitates a thorough understanding of various small intestine cancers. A multidisciplinary approach integrating clinical, radiological, and pathological expertise is essential for optimal patient care and achieving favorable outcomes in challenging cases, encompassing differential diagnosis, imaging findings, and surgical considerations.
